# Dosimetric comparison of two dose expansion methods in intensity modulated radiotherapy for breast cancer

**DOI:** 10.1186/s13014-023-02217-4

**Published:** 2023-02-04

**Authors:** Ran Tang, Aimin Li, Yingjing Li, Guanhua Deng, Yufeng Wang, Qing Xiao, Luosheng Zhang, Yue Luo

**Affiliations:** 1 Integrated Hospital of Traditional Chinese Medicine, Southern Medical University, No.13 Shiliugang Road, Guangzhou, 510315 Guangdong China; 2 Cancer Center, Southern Medical University, Guangzhou, 510315 China; 3 Guangdong 999 Brain Hospital, Guangzhou, 510510 China

**Keywords:** Breast cancer, Intensity modulated radiotherapy, Dose intensity expansion, Chest wall

## Abstract

**Background:**

To explore the dosimetric difference between IMRT-VB plan based on the establishment of external expansion structure and virtual bolus (VB) and IMRT-SF based on the skin flash (SF) tool of the Eclipse treatment planning system in postoperative chest wall target intensity modulation radiotherapy plan of breast cancer.

**Methods:**

Twenty patients with breast cancer were randomly selected as subjects to develop IMRT-VB plan based on virtual bolus and IMRT-SF plan based on skin flash tool of Eclipse treatment planning system. The planning target volume, monitor unit (MU) of every single treatment and the dosimetric parameters of organ at risk (OARs) were recorded. Paired t-test was used for normal distribution data while nonparametric paired Wilcoxon rank sum test was used for non-normal distribution data.

**Results:**

Both IMRT-VB and IMRT-SF plan can expand outward to the chest wall skin and meet the dose requirements of clinical prescription. The conformal index, the homogeneity index, D_2%_, D_98%_ and D_50%_ were significantly better in IMRT-SF plan than those in IMRT-VB plan (*P* < 0.05). The average MU of the IMRT-SF plan was much higher than that of the IMRT-VB plan (866.0 ± 68.1 MU vs. 760.9 ± 50.4 MU, *P* < 0.05). In terms of organ at risk protection, IMRT-SF plan had more advantages in the protection of ipsilateral lung and spinal cord than IMRT-VB plan (*P* < 0.05).

**Conclusion:**

Our study indicated that IMRT-SF plan displayed clinical application superiority compared to IMRT-VB plan, and the operation steps of which are simpler and faster. Besides, IMRT-SF plan took advantages in achieve effective external expansion of skin dose intensity and OARs protection.

## Background

Since the low-density lung tissue was in the irradiation area, the homogeneity index (HI) of traditional three-dimensional conformal radiotherapy is as high as 20% in the radiotherapy of breast cancer patients [1, 2]. Intensity modulated radiotherapy (IMRT) has been widely used in breast cancer radiotherapy recently. Compared with the traditional three-dimensional conformal radiotherapy, IMRT had obvious superiority in the uniformity and conformal degree of the target volume, as well as the protection of organs at risk. However, the target volume of the chest wall extends beyond the skin due to the movement of organs such as breathing and heartbeat [3, 4]. The existing radiotherapy planning system does not calculate the dose distribution outside the outer contour (that is, the skin), which was defaulted as zero-dose area. Although previous studies have reported two kinds of dose intensity expansion methods [5, 6], one is IMRT-VB plan based on virtual bolus (VB), the other is IMRT-SF based on the skin flash (SF) tool of the Eclipse treatment planning system, there were few reports on the application of these two dose expansion methods in postoperative chest wall IMRT planning of breast cancer. Here, by employing two different expansion methods in 20 patients with left breast cancer, we found that both IMRT-VB and IMRT-SF plan can expand outward to the chest wall skin and meet the dose requirements of clinical prescription. The conformal index (CI), the homogeneity index (HI), D_2%_, D_98%_ and D_50%_ were significantly better in IMRT-SF plan than those in IMRT-VB plan (*P* < 0.05). The average MU of the IMRT-SF plan was much higher than that of the IMRT-VB plan (866.0 ± 68.1 MU vs. 760.9 ± 50.4 MU, *P* < 0.05). In terms of organ at risk protection, IMRT-SF plan had more advantages in the protection of ipsilateral lung and spinal cord than IMRT-VB plan (*P* < 0.05). Taken together, our study indicated that IMRT-SF plan displayed clinical application superiority compared to IMRT-VB plan, and the operation steps of which are simpler and faster. Besides, IMRT-SF plan took advantages in achieve effective external expansion of skin dose intensity and OARs protection.

## Methods

### Human samples

Participant were a total of 20 patients with left breast cancer (pT3-4N0M0) who received postoperative radiotherapy in the Radiotherapy Center of Integrated Hospital of Traditional Chinese Medicine at Southern Medical University during August 2020 to December 2021. All patients were female, aged from 35 to 62 years old, with an average age of 50.40 ± 9.59 years old.

### CT simulation and target delineation

All patients were in supine position, with upper limbs abduction and arms crossed in front of forehead. The negative pressure vacuum pad was used for body position fixation. Patients were asked to maintain a steady breathing state during CT scanning. The upper boundary of CT scanning is at the level of cricothyroid membrane, the lower boundary is 5 cm below the fold of the lower edge of the breast with 0.5 cm scanning thickness. Philips Brilliance CT Big Bore was used to perform conventional CT simulation positioning scanning, with scanning slice thickness and slice spacing of 0.5 cm and resolution of 512 × 512.

The radiologist delineated the clinical target volume (CTV) of the chest wall according to the ICRU83 report and White J’s research [7, 8]. The planning target volume (PTV) expanded 5 mm on the basis of CTV, and the inner and posterior boundary was not allowed to extend to the lung [9]. The anterior boundary retracted in the subcutaneous 3 mm to form a structure named PTV_eval [10]. This PTV-eval is limited anteriorly to exclude the part that extends outside the body/patient and the first 3 mm of tissue under the skin in order to remove some of the buildup region for the DVH analysis. At the same time, heart, left lung, right lung, spinal cord and other organs at risk should be delineated. All the target areas and organs at risk were sketched by the same radiologist, and the prescription dose of PTV was 50 Gy/25F.

### Treatment planning

**Fig. 1:**
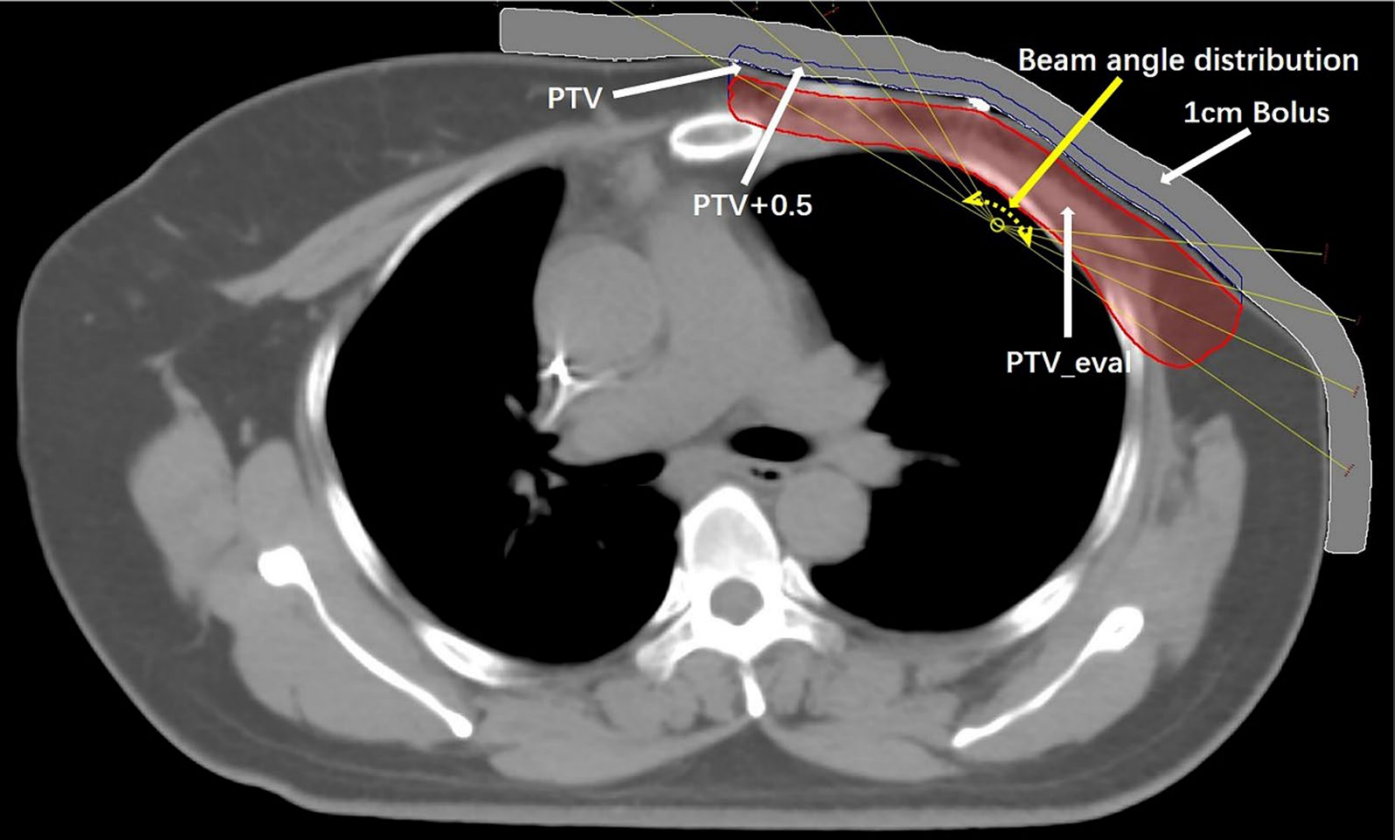
Schematic diagram of IMRT-VB and IMRT-SF plan designed for patients

The Eclipse13.6 planning system was used to establish the IMRT-VB plan based on the establishment of external expansion structure and virtual bolus (VB), and the IMRT-SF plan based on the skin flash (SF) tool of the Eclipse treatment planning system. The accelerator is Varian Clinic X, 6MV energy X-ray, the dose rate is 400MU/min, and the dose is calculated by analytical anisotropic algorithm (AAA), along with a grid size of 0.25 cm used for dose distribution computation. The intensity modulation plan of 8 fields was selected in both groups, and the field angle was based on a pair of tangent fields of breast target. In addition, 3 pairs of auxiliary tangent fields were added within the tangent field at an interval of 5–10° to form an intensity modulation plan of 8 fields. To better protect the normal tissue from exposure, the fixed jaw technique was used in the optimization of the plan [11], the field arrangement was set as previous reported [12]. Schematic diagram of IMRT-VB and IMRT-SF plan designed for patients was shown in Fig. 1.

IMRT-VB plan: Firstly, a 1.0 cm outer contour extension bolus was added to the breast part of the Body, and the CT value of the outer contour extension area was specified as 0HU. A new outer contour (“Body + bolus”) was generated by Boolean (union) operation between the original outer contour Body and the 1 cm virtual bolus. Then, a “PTV + 0.5” structure was generated by putting the PTV 0.5 cm outward toward the thorax. The angle layout of the radiation field was completed according to the above field layout principles. At the same time, fixed jaw techniques were used, and PTV and “PTV + 0.5” structures were simultaneously used as the target area optimization targets for flux optimization. After the flux optimization was completed and the clinical requirements were met, the virtual bolus was removed and the outer contour was reset to the original outer contour Body. The optimized radiation field flux was maintained for dose calculation. Therefore, the IMRT-VB plan for dose intensity expansion was obtained.

IMRT-SF Plan: The ‘Skin flash’ tool was a brush tool that extends the dose in the form of dose intensity projected at the inner edge of the field beyond the skin in the Beam Eyes View (BEV). The angle distribution and fixed jaw techniques of the radiation field were consistent with the IMRT-VB plan, and the PTV structure was taken as the target area optimization targets. After the flux optimization was completed and the clinical requirements were met, the ‘Skin flash’ tool in the Eclipse13.6 planning system was used to expand the skin flux of 0.5 cm in the chest wall target area of all the fields and then the dose distribution was calculated, and then the IMRT-SF plan was obtained.

### Plan evaluation and analysis

Dose volume histogram (DVH) was used to evaluate the exposure dose of target area and organ at risk. In the era of IMRT technology, the ICRU83 report recommends the use of IMRT technology, and the evaluation of the target no longer pays too much attention to the reported minimum and maximum dose points, but to the D_98%_ and D_2%_ indicators of the recommended target. Therefore, the specific parameters evaluated in this study include high-dose flat area (D_2%_), low-dose flat area (D_98%_), average dose (D_50%_), conformity index (CI) and homogeneity index (HI). The calculation formulas of CI and HI were as follows:

1 $${\text{CI}} = \frac{{V_{t,ref}^{2} }}{{{\text{V}}_{{\text{t}}} \times {\text{V}}_{{{\text{ref}}}} }}$$

2 $${\text{HI}} = \frac{{D_{2\% } - D_{{98{\% }}} }}{{D_{{50{\% }}} }}$$

wherein, ${ }V_{t,ref}$ means the volume covered by the prescription dose, $V_{t}$ means the target volume, $V_{ref}$ means the volume covered by the prescription dose in the target area, D_2%_, D_98%,_ D_50%_ represent the radiation dose received by 2%, 98% and 50% of the volume of the target, respectively. The closer the CI value is to 1, the better the dose suitability of the target area is; the closer the HI value is to 0, the more uniform the dose in the target area is. The evaluation parameters of organs at risk include V_5_%, V_10_%, V_20_%, V_30_% and D_mean_ of the left lung, V_5_%, V_10_% and D_mean_ of the right lung, V_5_% and D_mean_ of the heart, and D_max_ of the spinal cord.

### Plan verification

The flux of each beam of the two technology plans was collected by using the Portal Dosimetry function of the Clinac iX linear accelerator of Varian Company of the United States. Gamma analysis, a widely used method for evaluating relative dose contribution [13], was carried out using the standard of 3 mm/2%, and the passing rate was verified by statistical dose.

### Statistical analysis

The dosimetry parameters were analyzed by IBM SPSS25.0. The hypothesis test data were used to analyze whether it conformed to the normal distribution. The normal distribution data were shown as mean ± SD, and the non-normal distribution data were shown as M (Q1, Q3). Paired t-test was performed for normal distribution data analysis and nonparametric paired Wilcoxon rank sum test was used for non-normal distribution data analysis. *P* < 0.05 was considered as statistically significant.

## Results

### Target dose comparison

As shown in Table 1, the dosimetric indexes of CI, HI, D_2%_, D_98%_ and D_50%_ of IMRT-SF plan were significantly better than those of IMRT-VB plan (*P* < 0.05). In terms of monitor unit (MU), the average MU of the IMRT-SF plan was much higher than that of the IMRT-VB plan (866.0 ± 68.1 MU vs. 760.9 ± 50.4 MU, *P* < 0.05).

**Table 1:** Comparison of target dosimetry and MUs between two plans $\left( {\overline{x} \pm s} \right)$

Parameter	IMRT-SF	IMRT-VB	t-stat	*P*-value
CI	0.83 ± 0.03	0.68 ± 0.04	19.618	< 0.01
HI	0.07 ± 0.004	0.16 ± 0.01	− 26.010	< 0.01
D_2%_/cGy	5315.7 ± 20.1	5663.9 ± 34.3	− 30.826	< 0.01
D_98%_/cGy	4966.2 ± 9.3	4805.5 ± 52.0	9.822	< 0.01
D_50%_/cGy	5163.1 ± 13.5	5394.3 ± 25.3	− 31.262	< 0.01
MU	866.0 ± 68.1	760.9 ± 50.4	6.645	< 0.01

### Comparison of organs at risk

As shown in Table 2, compared with IMRT-VB plan, IMRT-SF plan had better dosimetric advantages in V_10_%, V_20_%, V_30_%, D_mean_ of left lung (*P* < 0.05). Moreover,

**Table 2:** Comparison of dosimetry of OARs between two plans $\left( {\overline{x} \pm s} \right)/M\left( {Q1, Q2} \right)$

Parameters	IMRT-SF	IMRT-VB	t/z-stat	*P*-value
Left lung V_5_/%	44.60 (43.53, 49.13)	44.75 (42.98, 48.93)	− 0.255	0.799
Left lung V_10_/%	30.72 ± 3.21	31.39 ± 2.99	− 2.831	0.02
Left lung V_20_/%	19.10 ± 1.91	19.64 ± 1.78	− 5.576	< 0.001
Left lung V_30_/%	12.86 ± 1.52	13.70 ± 1.56	− 7.517	< 0.001
Left lung D_mean_/cGy	1047.78 ± 83.38	1097.28 ± 77.94	− 8.016	< 0.001
Right lung D_mean_/cGy	109.94 ± 38.15	108.20 ± 35.71	1.341	0.213
Heart V_5_/%	34.14 ± 4.81	33.52 ± 4.64	0.804	0.442
Heart D_mean_	631.25 ± 80.07	635.76 ± 86.82	− 0.558	0.591
Cord D_max_/cGy	48.17 ± 6.08	48.78 ± 6.01	− 3.966	0.003
Right lung V_5_/%	2.30 (1.25, 3.43)	2.40 (0.93, 3.38)	− 0.475	0.635
Right lung V_10_/%	0 (0.00, 0.00)	0 (0, 0.10)	0.000	1.000

IMRT-SF plan exhibited better spinal cord protection than IMRT-VB plan (*P* = 0.003).

Besides, IMRT-SF plan showed comparable data in heart V_5_% (*P* = 0.442), Heart D_mean_ (*P* = 0.591), V_5_% of left lung (*P* = 0.799) and V_5_%, V_10_%, D_mean_ of right lung relative to IMRT-VB plan (*P* = 0.635, 1.000, 0.213, respectively).

### Patient-specific QA results

**Fig. 2:**
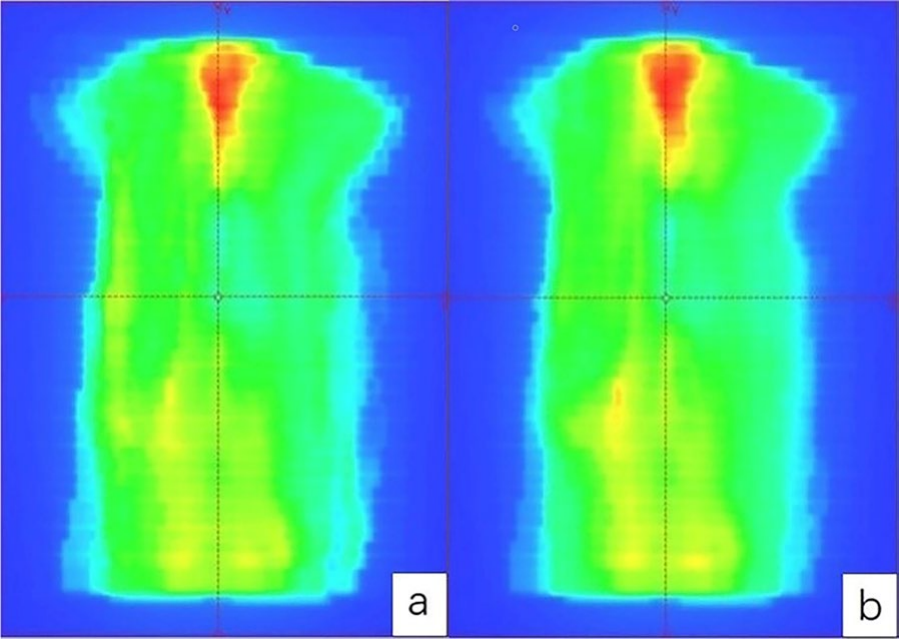
Flux map of actual collection of EPID of two plans for the same patient, **a** IMRT-SF plan, **b** IMRT-VB plan

**Fig. 3:**
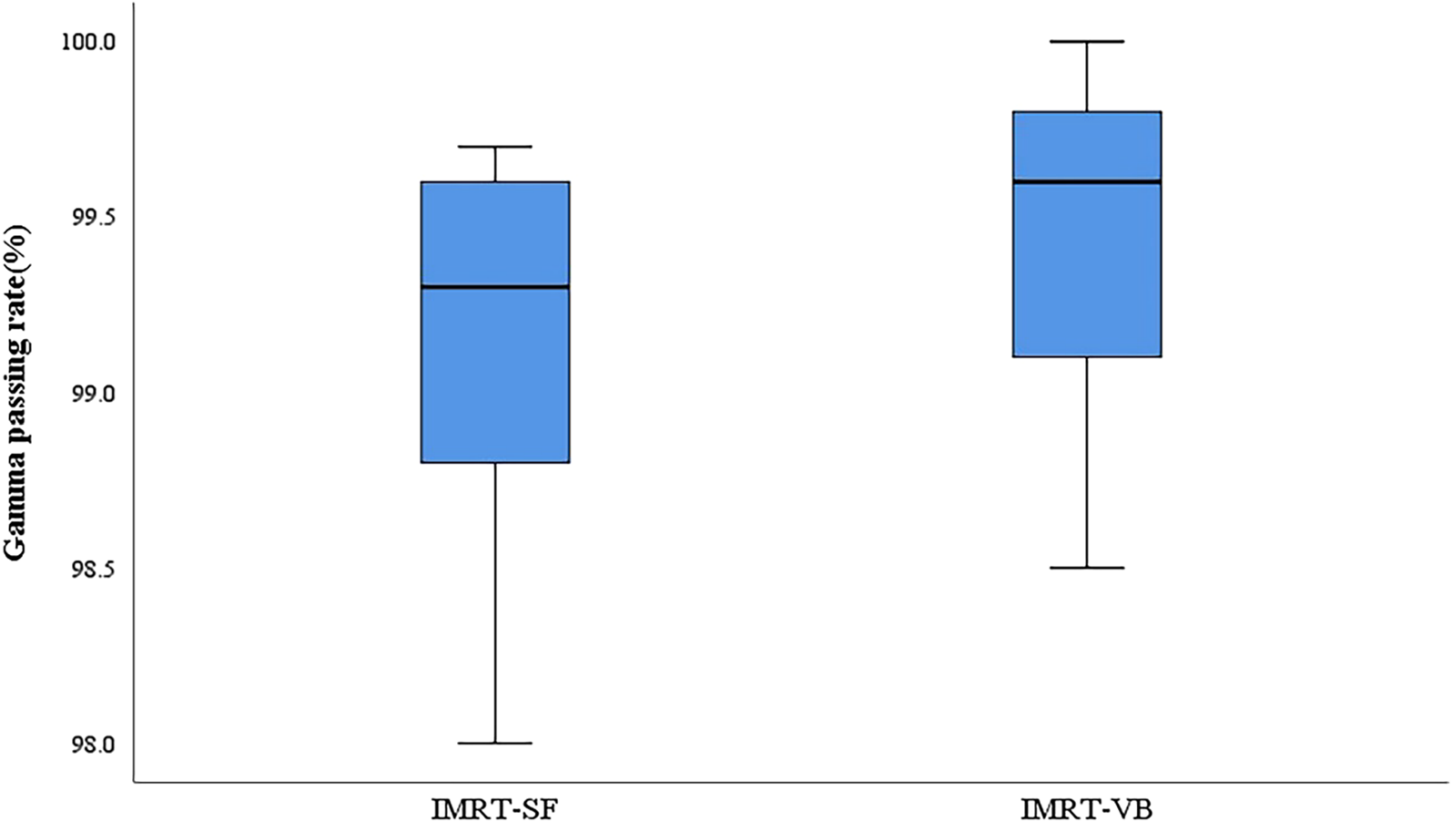
For the 3%/2 mm evaluation criteria, gamma passing rate

As shown in Figs. 2 and 3, the result showed that the gamma passing rate of IMRT-SF plan was 99.16 ± 0.54%, and that of IMRT-VB plan is 99.48 ± 0.46%. The passing rate of IMRT-SF plan is slightly lower than that of IMRT-VB plan (t = − 9.798, *P* < 0.0001).

## Discussion

Many studies have shown that the intensity modulated radiotherapy (IMRT) mainly in the tangent field of postoperative radiotherapy of breast cancer, can not only improves the dose uniformity, but also reduces the radiation dose to the lung, spinal cord and other organs at risk [14, 15]. In order to compensate for the target area movement caused by organ movement and positioning errors, the target area of breast cancer generally expands CTV by 5–10 mm as PTV, which makes the PTV in the chest wall area expand directly outside the body. Since the photon dosimetry possess the characteristics of dose building area, the IMRT plan optimization process will continuously increase the dose of the skin and the area outside the skin, resulting in unreasonable optimization results and even failed plan. In response to the above problems, the ICRU 62 report and other scholars have proposed some relevant solutions [5, 16, 17]. Sankar et al. [2] used the ‘skin flash’ tool of Varian eclipse planning system for dose expansion to effectively exteriorize skin flux to meet clinical therapeutic requirements. Consistent with previous studies reported by Chopra [18] and Morrow [19], the flux optimization results of the IMRT-VB and IMRT-SF plans in this study have realized the dose intensity expansion of 0.5 cm towards the thorax, which effectively solves the problem of insufficient dose and off-target effects in the chest wall target area caused by respiratory movement.

Patients with breast cancer undergoing postoperative radiotherapy may miss the target in the actual treatment process due to the thickness of their chest wall and respiratory motility, leading to insufficient actual radiation dose to the target area of the chest wall [20, 21]. In this study, for patients receiving chest wall target radiotherapy, two different dose intensity expansion methods of radiotherapy plans were designed using eight field IMRT technology with tangent field. The conformal index (CI), the homogeneity index (HI), D_2%_, D_98%_ and D_50%_ were significantly better in IMRT-SF plan than those in IMRT-VB plan (*P* < 0.05). The average MU of the IMRT-SF plan was much higher than that of the IMRT-VB plan (866.0 ± 68.1MU vs. 760.9 ± 50.4MU, *P* < 0.05). In terms of organ at risk protection, IMRT-SF plan had more advantages in the protection of ipsilateral lung and spinal cord than IMRT-VB plan (*P* < 0.05). However, default values of the "Skin flash" tool were adopted in the IMRT-SF plan, whether the adjustment of the default parameters has an impact on the total monitor unit needs to be further studied. Giorgia et al. [22] assigned soft-tissue equivalent HU to its artificial expansion. According Giorgia N’s research, the virtual bolus was specified as 0 HU in our IMRT-VB plan as it is much closer to human muscle and adipose tissue. However, Ugurlu et al. [23] specified the HU value of virtual bolus as − 700, while Thilmann et al. [24] specified the HU as − 60. Since there were different choice of the HU values of the virtual bolus, the most appropriate HU value needs to be further explored and whether the changes of the HU value would affect the total monitor units still needs to be further studied.

It has been reported that adding effective bolus can increase the skin surface dose of photon rays with 6MV energy from 10 to 40% to nearly 100% [25]. However, for patients with no skin invasion, the skin surface dose level does not need to reach the 100% dose level. Once the bolus is added, it may aggravate acute skin injury, interrupt the treatment, and then increase the risk of chest wall recurrence. Studies [26, 27] found that bolus was unable to reduce the recurrence rate of chest wall and improve the survival rate. Lizondo [3] found that a 1 cm bolus thickness equal to the CTV-PTV margin plus 5 mm. Therefore, in our study, the IMRT-VB plan uses a 1 cm virtual bolus to achieve the purpose of dose intensity expansion, and the virtual bolus is removed in the final dose volume calculation stage, which could not only achieve the purpose of dose intensity expansion, but also has a certain protective effect on the skin. Even so, the dose level (D_2%_) of IMRT-VB plan in the high dose hot zone of the target area is still slightly higher than that of IMRT-SF plan. The AAPM TG218 report [28] pointed out that it is too sweeping to adopt a dose distance error standard of 3%/3 mm γ analysis criteria in the clinical IMRT plan validation analysis. Therefore, the more critical γ Analytical standard (3%/2 mm) was employed in our study. Although the experimental results show that the γ passing rate of 99.16 ± 0.54% in IMRT-SF plan is slightly lower than that of 99.48 ± 0.46% in IMRT-VB plan (t = − 9.798, *P* < 0.0001), the gamma passing rate both exceeded 95%, indicating that both plans met the clinical treatment requirements. Although this study revealed the dosimetric effects of IMRT-SF and IMRT-VB dose expansion methods on target area irradiation of chest wall and organs at risk after breast cancer surgery, there are still some limitations, research samples amplification and multi-center validation were needed for further exploration.

## Conclusion

In general, our study indicated that IMRT-SF plan displayed clinical application superiority compared to IMRT-VB plan, and the operation steps of which are simpler and faster. Besides, IMRT-SF plan took advantages in achieve effective external expansion of skin dose intensity and OARs protection.

## Data Availability

The data that support the findings of this study are available from the corresponding author upon reasonable request.

## Abbreviations

IMRT: Intensity modulated radiotherapy
VB: Virtual bolus
SF: Skin flash
PTV: Planning target volume
MU: Monitor unit
OARs: Organ at risk
CI: Conformal index
HI: Homogeneity index
CTV: Clinical target volume
AAA: Analytical anisotropic algorithm
BEV: Beam eyes view
DVH: Dose volume histogram
VMAT: Volume intensity modulated arc therapy

## References

[1] Ikner CL, Russo R, Podgorsak MB, Proulx GM, Lee RJ (1998). Comparison of the homogeneity of breast dose distributions with and without the medial wedge. Med Dosim Off J Am Assoc Med Dosim.

[2] Sankar A, Velmurugan J (2009). Different intensity extension methods and their impact on entrance dose in breast radiotherapy: a study. J Med Phys.

[3] Lizondo M, Latorre-Musoll A, Ribas M, Carrasco P, Espinosa N, Coral A (2019). Pseudo skin flash on VMAT in breast radiotherapy: optimization of virtual bolus thickness and HU values. Phys Med.

[4] Sick J, Fontenot J (2018). The air out there: treatment planning when target volumes extend beyond the skin. Int J Radiat Oncol Biol Phys.

[5] Chang J, Baker J (2018). Consider using virtual bolus. Int J Radiat Oncol Biol Phys.

[6] Dyer BA, Jenshus A, Mayadev JS (2019). Integrated skin flash planning technique for intensity-modulated radiation therapy for vulvar cancer prevents marginal misses and improves superficial dose coverage. Med Dosim.

[7] White J (2015). Defining target volumes in breast cancer radiation therapy for the future: back to basics. Int J Radiat Oncol Biol Phys.

[8] Hodapp N (2012). The ICRU report 83: prescribing, recording and reporting photon-beam intensity-modulated radiation therapy (IMRT). Strahlenther Onkol.

[9] Vargo JA, Beriwal S (2015). RTOG chest wall contouring guidelines for post-mastectomy radiation therapy: Is it evidence-based?. Int J Radiat Oncol Biol Phys.

[10] Mamounas EP, White JR, Bandos H, Julian TB, Kahn AJ, Shaitelman SF (2014). NSABP B-51/RTOG 1304: randomized phase III clinical trial evaluating the role of postmastectomy chest wall and regional nodal XRT (CWRNRT) and post-lumpectomy RNRT in patients (pts) with documented positive axillary (Ax) nodes before neoadjuvant chemotherapy (NC) who convert to pathologically negative Ax nodes after NC. J Clin Oncol.

[11] Wang JQ, Yang ZZ, Hu WG, Chen Z, Yu XL, Guo XM (2017). Intensity modulated radiotherapy with fixed collimator jaws for locoregional left-sided breast cancer irradiation. Oncotarget.

[12] Huang JH, Wu XX, Lin X, Shi JT, Ma YJ, Duan S (2019). Evaluation of fixed-jaw IMRT and tangential partial-VMAT radiotherapy plans for synchronous bilateral breast cancer irradiation based on a dosimetric study. J Appl Clin Med Phys.

[13] Kunii Y, Tanabe Y, Nakamoto A, Nishioka K (2022). Statistical analysis of correlation of gamma passing results for two quality assurance phantoms used for patient-specific quality assurance in volumetric modulated arc radiotherapy. Med Dosim Off J Am Assoc Med Dosim.

[14] Pignol JP, Truong P, Rakovitch E, Sattler MG, Whelan TJ, Olivotto IA (2016). Ten years results of the Canadian breast intensity modulated radiation therapy (IMRT) randomized controlled trial. Radiother Oncol.

[15] Schubert LK, Gondi V, Sengbusch E, Westerly DC, Soisson ET, Paliwal BR (2011). Dosimetric comparison of left-sided whole breast irradiation with 3DCRT, forward-planned IMRT, inverse-planned IMRT, helical tomotherapy, and topotherapy. Radiother Oncol.

[16] Balter P, Nitsch P (2018). Consider re-simulation or evaluate with and without density overrides. Int J Radiat Oncol Biol Phys.

[17] Price M, Yock A (2018). Employ piece-wise optimization. Int J Radiat Oncol Biol Phys.

[18] Chopra S, Dinshaw KA, Kamble R, Sarin R (2006). Breast movement during normal and deep breathing, respiratory training and set up errors: implications for external beam partial breast irradiation. Br J Radiol.

[19] Morrow NV, Stepaniak C, White J, Wilson JF, Li XA (2007). Intra- and interfractional variations for prone breast irradiation: an indication for image-guided radiotherapy. Int J Radiat Oncol Biol Phys.

[20] Buwenge M, Cammelli S, Ammendolia I, Tolento G, Zamagni A, Arcelli A (2017). Intensity modulated radiation therapy for breast cancer: current perspectives. Breast Cancer (Dove Medical Press).

[21] Richter A, Sweeney R, Baier K, Flentje M, Guckenberger M (2009). Effect of breathing motion in radiotherapy of breast cancer 4D dose calculation and motion tracking via EPID. Strahlenther Onkol.

[22] Giorgia N, Antonella F, Alessandro C, Eugenio V, Luca C (2011). Planning strategies in volumetric modulated arc therapy for breast. Med Phys.

[23] Ugurlu TB, Koksal Akbaş C, Ibis K, Becerir HB (2022). The effect of using virtual bolus on VMAT plan quality for left-sided breast cancer patients. Appl Radiat Isot.

[24] Thilmann C, Grosser KH, Rhein B, Zabel A, Wannenmacher M, Debus J (2002). Virtual bolus for inversion radiotherapy planning in intensity-modulated radiotherapy of breast carcinoma within the scope of adjuvant therapy. Strahlenther Onkol.

[25] Soong IS, Yau TK, Ho CM, Lim BH, Leung S, Yeung RM (2004). Post-mastectomy radiotherapy after immediate autologous breast reconstruction in primary treatment of breast cancers. Clin Oncol (R Coll Radiol).

[26] Hui Z, Li Y, Yu Z, Liao Z (2006). Survey on use of postmastectomy radiotherapy for breast cancer in China. Int J Radiat Oncol Biol Phys.

[27] Vu TT, Pignol JP, Rakovitch E, Spayne J, Paszat L (2007). Variability in radiation oncologists' opinion on the indication of a bolus in post-mastectomy radiotherapy: an international survey. Clin Oncol (R Coll Radiol).

[28] Miften M, Olch A, Mihailidis D, Moran J, Pawlicki T, Molineu A (2018). Tolerance limits and methodologies for IMRT measurement-based verification QA: recommendations of AAPM task group No. 218. Med Phys.

